# Influence of Family Social Support and Diabetes Self-Efficacy on the Emotional Wellbeing of Children and Adolescents with Type 1 Diabetes: A Longitudinal Study

**DOI:** 10.3390/children10071196

**Published:** 2023-07-10

**Authors:** Joaquín Villaécija, Bárbara Luque, Rosario Castillo-Mayén, Naima Z. Farhane-Medina, Carmen Tabernero

**Affiliations:** 1 Department of Psychology, University of Cordoba, 14071 Cordoba, Spain; jvillaecija2@uco.es (J.V.); bluque@uco.es (B.L.); rcmayen@uco.es (R.C.-M.); z62famen@uco.es (N.Z.F.-M.); 2 Maimonides Biomedical Research Institute of Cordoba (IMIBIC), 14004 Cordoba, Spain; 3 Instituto de Neurociencias de Castilla y León (INCYL), University of Salamanca, 37007 Salamanca, Spain; 4 Department of Social Psychology and Anthropology, University of Salamanca, 37005 Salamanca, Spain

**Keywords:** type 1 diabetes, self-efficacy, emotional subjective wellbeing, self-care, family social support, children, adolescents, explanatory model, mediation

## Abstract

Type 1 diabetes (T1D) is a chronic disease that is usually diagnosed in childhood, underscoring the importance of early disease control for overall wellbeing. Our aim was to design an explanatory model of subjective emotional wellbeing in children and adolescents with T1D. A longitudinal study was conducted at the Reina Sofia University Hospital in Cordoba (Spain). A total of 151 patients (mean age = 14.50, *SD* = 2.67; 41.1% girls) participated at T1, while 97 participated at T2 (mean age = 14.93, *SD* = 2.56; 39.2% girls). Participants completed a self-report questionnaire. Descriptive, reliability, correlation, path, and mediation analyses were performed. The explanatory model showed excellent fit indices [χ^2^ (10) = 8.62, *p* = 0.57, RMSEA = 0.00, 95% [0.00, 0.10], CFI = 1.00, GFI = 0.98, AGFI = 0.93, and TLI = 1.01]. The results showed significant and positive relationships between family social support and subjective emotional wellbeing and improved self-care skills. Self-efficacy presented a mediating role between family social support and subjective emotional wellbeing. Given that self-efficacy is a self-regulatory mechanism and a determinant of health, it is argued that future psychoeducational interventions could aim to improve self-efficacy to manage chronic diseases, to achieve greater emotional wellbeing in children and adolescents with T1D.

## 1. Introduction

Type 1 diabetes (T1D) is estimated to have a prevalence of 8.4 million cases worldwide, 18% of which are in people under 20 years of age, a figure that could double by 2040 [1]. It is a chronic disease characterized by insulin deficiency due to the attack and destruction of insulin-producing cells in the pancreas [2,3]. Its rapid growth in recent years provides evidence of an intersectionality between different factors, including environmental, which in some ways could explain this rise [4,5]. In Spain, it is estimated that between 11 and 24 children under 15 years of age out of every 100,000 currently have T1D; it is not possible to obtain an exact figure due to the methodological and regional heterogeneity of studies [6]. The fact that the diagnosis occurs mainly at a young age means that people with this disease live most of their lives with it. Living more years with a disease can be synonymous with a higher risk of health complications, but it can also be an important opportunity to re-educate the patient in behaviors that can lead to a better quality of life. For this reason, we focused our efforts on understanding the role of psychosocial variables in this population, to identify the guidelines to achieving their wellbeing.

It is not easy to find an explicit definition of wellbeing, and there is no consensus among the scientific community on the term and others related to it. Recently, a paper has compiled many these terms and their definitions, showing the complexity of the issue [7]. What is clear is that from a mental-health approach, the wellbeing each person perceives for themselves is still a subjective construct. In this case, the correct term to use is subjective wellbeing. A well-established definition of subjective wellbeing (SWB); although its author recognizes that it could certainly be determined by multiple factors; is the positive self-evaluation, both cognitive and affective, that an individual makes of their life at a given moment [8]. In our case, we will focus on the emotional aspect of wellbeing. Emotional subjective wellbeing (ESW) will be understood as the affective evaluation of emotional balance, one of the many components that other studies have already considered as part of the term [9].

One of the most recent contributions to this concept contemplates the importance of social relationships and social support as a fundamental factor of ESW [10]. This contribution is in line with other studies, thus Turner [11] affirmed that the relationship between social support and wellbeing is especially relevant in stressful situations, which allows us to believe that it could play a fundamental role in the management of a chronic disease, such as T1D. Even in the general population, social support has been shown to act as a predictor of subjective wellbeing, especially family social support [12]. In adult patients with diabetes, a higher level of perceived social support was associated with a lower level of diabetes distress and other depressive symptoms [13]. These contributions open the door to further exploring the role of social support perceived by children and adolescents with T1D, and its relationship with SWB.

Based on these findings, we hypothesized for a clinical population of children and adolescents with T1D:

**H1.**  *Family social support is positively related to emotional wellbeing.*

**H2.**  *Family social support is positively related to the social support of one’s friends and significant other.*

Social networks are important for health not only because of the emotional support they generate, but also because of the sense of belonging and identity, and social facilitation of acceptance of normative behaviors, that they provide [14]. In this sense, although previous research has found that social support can predict self-care behaviors in people with diabetes [15], this fact does not seem to be as clear in T1DM. A meta-analysis in this regard highlights the need to focus more research toward testing how social support influences self-care in this population [16]. In this sense, self-care can be considered as a self-regulatory mechanism of behavior, linked to the concept of self-efficacy. Not surprisingly, self-efficacy is defined as the personal belief in one’s own ability to perform a specific task at a specific time, with a satisfactory goal [17,18], whether in diet, exercise, or any other area associated with self-care or other behaviors. In this regard, numerous investigations have shown that self-efficacy is an important factor in behavior modification in children and adolescents [19]. Bandura [17,18] proposed that, in addition to from one’s own experience, one can develop self-efficacy judgments from the beliefs of others that are manifested through the social support received, whether from family, friends, or medical teams. Therefore, the social support received is a source of the construction of the self-efficacy judgment that individuals have about the management of the disease, a fact that has already been proven along patients with diabetes when evaluating the support received from healthcare professionals [20]. Similarly, a positive emotional state could be related to greater confidence in one’s own ability to create the necessary resources to manage the disease over time; i.e., greater self-efficacy and, consequently, better health [21,22].

Hence, research suggests that fostering environments that increase the confidence of young people with T1D in their own abilities to manage the disease could benefit their own behaviors [23]. Previous studies have already demonstrated the importance of social support from primary caregivers in the health of adolescents with diabetes, and the positive impact that this could have on their own disease management [24]. Parental involvement in disease management is the earliest support that minors and adolescents receive, support that remains equally necessary with increasing age [25]. In the case of adolescents, some studies suggest the importance of considering self-efficacy both in the regulation of negative emotions, which would facilitate their coping with their life goals [26,27]; and in improving the wellbeing of young people [28]. Along the same lines, very recent studies show that in endocrinological patients with diabetes, the level of self-efficacy that patients have in disease control can be a predictor of perceived wellbeing and quality of life [29,30]. Thus, for good reason, self-efficacy is presented as the key element of psychoeducational processes of young patients with T1DM in numerous studies [31,32,33]. All this suggests the importance of enhancing self-efficacy judgments in affective regulation, for the improvement of health in adolescents with T1D [34].

Therefore, we hypothesize:

**H3.**  *Family social support is positively related to, and acts as a predictor of, self-efficacy in managing T1D.*

**H4.**  *Subjective emotional wellbeing is positively related to, and acts as a predictor of, positive outcome expectations about health.*

**H5.**  *Self-efficacy in managing T1D is positively related to, and acts a predictor of, emotional subjective wellbeing.*

**H6.**  *Self-efficacy in T1D acts as a mediating variable between family social support and perceived emotional wellbeing.*

Considering the above, the main objective of this study was to design an explanatory model of subjective emotional wellbeing in a population of children and adolescents with T1D. As a secondary objective, we tested the mediating role of self-efficacy in managing T1D in the relationship between the family social support and emotional wellbeing of these patients. As a final hypothesis, we expected that the variables assessed would not demonstrate a significant change over time.

## 2. Materials and Methods

### 2.1. Study Sample

The inclusion criteria for participant selection were patients diagnosed with T1D between 10 and 19 years old, who attended regular follow-up appointments with the medical team. These patients had to be native speakers of, or fluent in, Spanish. Patients outside of these ages were excluded, as well as those who had attended for a reason other than T1D. The clinical sample included a total of 151 pediatric and young patients with T1D at T1, randomly selected from the endocrinology unit of the Reina Sofia University Hospital of Cordoba (Spain), and 97 at T2.

### 2.2. Procedure

Following the approval of the project by the Cordoba Research Ethics Committee (date and version of the protocol: 1–6 January 2020; committee reference: 5166; report no. 327; approved on 28 September 2021), the research team visited the endocrinology units at the Reina Sofía University Hospital in Cordoba (Spain) to inform the parents about the project’s objectives, and to invite them to participate, although they had been previously informed by the medical team. They were informed that participation was completely anonymous and voluntary, and that once they had started, they could drop out at any time, if they felt it was necessary. The data collection took place on the day the patients attended their regular check-up, and after the signing of the informed consent form by the parent accompanying the minor, or by the patient if they were of legal age. Subsequently, a questionnaire was administered using an online platform (Questback Unipark, Cologne, Germany), and was completed using the tablets provided by our laboratory. A researcher was always present to address any inquiries that arose during the completion of the questionnaire. Usually, children under 14 years of age needed help from the researcher, while those older than 14 years completed the questionnaire autonomously. The entire process took approximately 25 min. The data were collected at T1 (since December 2021), and T2 (since March 2022). Between T1 and T2, 3 months elapsed in the case of pediatric patients (up to 14 years of age), and 6 months in the case of older adolescents (the time interval in which they had attended medical check-ups). Although it was a brief period, and the patients were not subjected to any type of intervention beyond a routine medical follow-up, a longitudinal design was carried out, to give consistency to the exploratory model and the mediation analysis, considering that the variables are maintained over time.

### 2.3. Measures

#### 2.3.1. Demographic and Clinical Data

The patients were asked to provide information regarding their age, sex, and age at disease onset. These data, together with the duration of illness (in years), insulin regimen, time in range, and glycosylated hemoglobin (Hba1c), were subsequently cross-checked with the clinical reports provided by the medical team.

#### 2.3.2. Perceived Social Support

The Multidimensional Scale of Perceived Social Support (MSPSS) was used to measure the subject’s perceived social support [35]. The scale presents three factors according to the agent in which the support is perceived: Family (e.g., “My family really tries to help me”), Friends (e.g., “I can talk about my problems with my friends”), and Significant Other (e.g., “I have a special person who is a real source of comfort to me”). For our study, we used a version validated for the Spanish adolescent population [36]. In the original version the scale presented a Cronbach’s alpha of 0.88, while in the validation, it was 0.89. For factors, the original and validated versions, respectively, showed: α = 0.87 and α = 0.85 (Family); α = 0.85 and α = 0.89 (Friends); α = 0.91 and α = 0.79 (Significant Other). In our study, in the total scale α = 0.92, while the factors showed 0.90, 0.89, and 0.86, respectively. Participants answered 12 items on a five-point Likert scale (1 = “strongly disagree”; 5 = “in full agreement”) to the question “To what extent do the following statements describe you?”.

#### 2.3.3. Emotional Subjective Wellbeing

The emotional subjective wellbeing was assessed using a shortened version of the Positive Affect and Negative Affect Scale (PANAS-SF) [37]. In addition to positive and negative affect, studies have already used it to measure the emotional balance between positive affect and negative affect (subtracting scores), obtaining a measure of the emotional part of subjective or perceived wellbeing [38]. Specifically, the Spanish validation of the PANAS-SF was used [39]. This scale is composed of 10 items, organized in two factors of five each: positive affect (PA) and negative affect (NA). The original short version presented good internal consistency: α = 0.86 (PA) and α = 0.82 (NA); the validated Spanish version for children showed α = 0.77 for PA and α = 0.79 for NA; while with our sample, Cronbach’s alpha was 0.92 at T1, and 0.90 at T2, to the emotional balance variable. Participants answered on a five-point Likert scale (1 = “not at all”; 5 = “totally”) to the statement “Identify to what extent each word reflects how you feel about your life in general”.

#### 2.3.4. Positive Outcome Expectations of Type 1 Diabetes Self-Management

The positive outcome expectations of T1D self-management were assessed using a factor of the shortened version of the Outcome Expectations of Diabetes Self-Management scale (OEDM) [40]. We only used the dimension of positive expectations (OEDM-P), discarding the negative ones. OEDM-P had a Cronbach’s alpha in the original study of 0.84. For our study, we needed to adapt the instrument. First, two items related to economic savings were eliminated (“Save me money now” and “Save me money in the future”). This decision was taken because the Spanish population has its health needs guaranteed by the state, free of charge. Then, after performing an exploratory factor analysis (EFA), we decided to organize it into three factors: social activity (e.g., “Make me be admired by my friends”); energy and sport (e.g., “Make me better in sports”); and self-management for health (e.g., “Keep my diabetes in better control”). The item “Make me have fewer high blood sugars” had to be eliminated, due to its low factorial weight. Cronbach’s alphas were 0.73, 0.72, and 0.76, respectively (0.79 one-factor). Participants answered on a five-point Likert scale (1 = “not sure at all”; 5 = “absolutely sure”) to the statements that continued the affirmation “If I did everything I had to do to take care of my diabetes…”.

#### 2.3.5. Self-Efficacy in Type 1 Diabetes Management

The self-efficacy in T1D management was assessed using a shortened version of the Self-Efficacy for Diabetes Self-Management scale (SEDM-SF) [40]. This 10-item unifactorial instrument, already used in the child and adolescent population, was translated, and validated to the Spanish population by the research team (currently under publication). Participants answered items on a five-point Likert scale (1 = “not sure at all”; 5 = “absolutely sure”) to the question “How confident do you feel about doing the following things well?”. Two examples of items are: “Choose healthy foods when you go out to eat” and “Manage your diabetes the way your health care team wants you to”. The Cronbach’s alpha was good (α = 0.90 in the original study). For our sample, Cronbach’s alpha was 0.85.

### 2.4. Data Analysis

Firstly, descriptive analyses were conducted for the sociodemographic data of the sample, and Cronbach’s α was calculated to assess the internal consistency of the instruments. Secondly, we explored the relationships between the variables included in the study using Spearman’s correlation test, after rejecting the null hypothesis using the Kolmogorov–Smirnov (*K-S*) test, and concluding that the data did not show a normal distribution. Thirdly, regression, repeated measures *t*-tests, and path analysis were conducted to understand the direction of these relationships, and to create an explanatory model. All analyses were performed using the Statistical Package for Social Sciences (SPSS.25), except for the path analysis, for which Amos24 was used. The model fit was assessed with the chi-square statistic (χ^2^), the goodness-of-fit index (GFI), the adjusted goodness-of-fit index (AGFI), the comparative-fit index (CFI), the root–mean–square error of approximation (RMSEA), and the Tucker–Lewis index (TLI), following the recommendations of Schermelleh-Engel and collaborators [41]. An adequate model fit should show GFI and TLI values greater than 0.95, CFI values greater than 0.97, AGFI values greater than 0.90, and RMSEA values lower than 0.05 [41]. Lastly, mediation analysis was performed with the fourth model of PROCESS for SPSS macro [42]. A confidence interval of 95% and 10,000 bootstrap resamples was executed.

## 3. Results

### 3.1. Preliminary Analyses

Once the demographic and clinical data of the sample were described (see Table 1), the internal consistency of the instruments was verified.

Then, a descriptive analysis and Spearman’s correlation test for studied variables were performed. Table 2 and Table 3 show these data. All correlations were in the expected direction. The self-efficacy for the management of T1D was positively related to positive outcome expectations of T1D self-management (and to factors of social activity and self-management for health), perceived social support (and for all its factors), and emotional subjective wellbeing at T1. Moreover, emotional subjective wellbeing was positively related to age, positive outcome expectations of T1D self-management (and for all its factors), and perceived social support (and for its factors) at T1. These correlations were repeated at T2.

To analyze changes over time for all variables studied, repeated measures or paired-sample *t*-tests were performed for each variable. In accordance with our hypotheses, most variables did no exhibit significant changes, specifically the short-form self-efficacy for diabetes self-management [*t* (96) = 0.67, *p* = 0.50, Cohen’s *d* = 0.55], the positive outcome expectations for diabetes self-management of social activity [*t* (96) = 1.16, *p* = 0.25, Cohen’s *d* = 1.05], the family social support [*t* (96) = −0.09, *p* = 0.93, Cohen’s *d* = 0.58], the social support from friends [*t* (96) = 1.70, *p* = 0.09, Cohen’s *d* = 0.60], the significant other social support [*t* (96) = 0.35, *p* = 0.73, Cohen’s *d* = 0.58], and the emotional subjective wellbeing [*t* (96) = −0.25, *p* = 0.81, Cohen’s *d* = 0.99]. As can be seen in Table 2 and Table 3, the changes in the means of each factor are minor. However, the values of the following variables were significantly reduced: positive outcome expectations for diabetes self-management of health [*t* (96) = 2.56, *p* = 0.01, Cohen’s *d* = 0.73], and positive outcome expectations for energy and sport [*t* (96) = 2.26, *p* = 0.03, Cohen’s *d* = 0.99].

### 3.2. Explanatory Model

**Figure 1:**
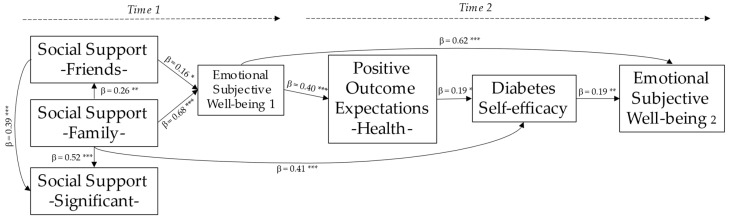
Explanatory model for self-efficacy regarding disease management and subjective wellbeing in children and adolescents with T1D. Standardized parameter estimates. * *p* < 0.05; ** *p* < 0.01; *** *p* < 0.001.

An explanatory model was developed to examine the impact of perceived social support (family, friends, and significant other) on self-efficacy regarding T1D management, positive outcome expectations for self-management of health, and emotional subjective wellbeing, in a population of children and adolescents with T1D. The results showed that the proposed explanatory model was well-fitted to the data: χ^2^ (10) = 8.62, *p* = 0.57, CMIN = 0.86, RMSEA = 0.00, 95% [0.00, 0.10], CFI = 1.00, GFI = 0.98, AGFI = 0.93, and TLI = 1.01. The standardized parameter estimates are reported in Figure 1.

The path analysis revealed significant findings. Family social support had a direct influence on both friends’ social support and significant other social support, while friends’ social support also influenced significant other social support. Moreover, both friends’ social support and family social support had a direct impact on emotional subjective wellbeing. Additionally, subjective wellbeing emerged as a predictor variable for positive outcome expectations regarding health self-management. This, in turn, positively predicted self-efficacy in diabetes control. Additionally, family social support showed a direct association with diabetes self-efficacy, which subsequently contributed to improved emotional subjective wellbeing.

### 3.3. Mediation Analysis

**Figure 2:**
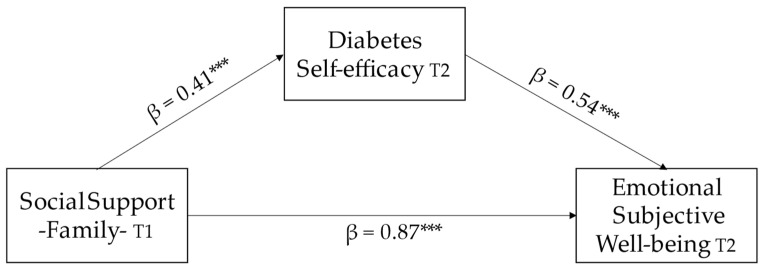
Mediation analysis, where diabetes self-efficacy at T2 acts as a mediating variable between the relationship of family social support and emotional subjective wellbeing, in children and adolescents with T1D. *** *p* < 0.001.

The PROCESS model number 4 was conducted, with a confidence interval of 95% and 10,000 bootstrap resamples, to check the mediating of diabetes self-efficacy, as shown by the indirect effect, between the relationship of family social support and emotional subjective wellbeing (β = 0.23, SD = 0.09, 95% CI [0.09, 0.44]). Although the regression between family social support and wellbeing holds without the mediation of diabetes self-efficacy, its strength diminishes (β = 0.65, *p* < 0.01). See Figure 2.

## 4. Discussion

The main aim of this study was to longitudinally design an explanatory model of emotional subjective wellbeing in a population of children and adolescents with T1D. The psychosocial variables included were social support (family, friends, and significant other), positive outcome expectations, self-management for health, self-efficacy for managing T1D, and emotional subjective wellbeing.

Regarding the resulting explanatory model, our study shows some findings in relation to the interactions that occur between the included variables. These findings of relevance have important practical implications for infantile–juvenile patients with T1D: (a) both social support from family and social support from friends play pivotal roles in emotional subjective wellbeing (H1 confirmed); (b) family social support plays a fundamental role in shaping the perception of support from friends, and both family and friends’ support is important in determining the perception of support from a significant other (H2 confirmed); (c) family social support is a significant predictor of diabetes self-efficacy, and it plays a crucial role in enhancing self-efficacy in disease management (H3 confirmed); (d) a higher level of subjective wellbeing is positively associated with an increased propensity for setting positive goals related to health management (H4 confirmed); and, (e) having a positive belief about disease control is associated with a heightened perception of ESW (H5 confirmed). In relation to the mediation analysis, the great weight that self-efficacy for managing T1D has in this population is confirmed, and we can say that, although family social support can act as a predictor variable of the emotional wellbeing of children and adolescents with T1D, it is diabetes self-efficacy that allows this prediction to occur (H6 confirmed).

Considering our findings, along with those reported in previous studies, it can be concluded that social support plays a fundamental role in predicting self-care behaviors and, therefore, disease management, among these patients [15,43]. In particular, our explanatory model underscores the significance of family social support in fostering an enhanced self-efficacy for disease control, a finding in line with other studies [24]. Not only that, but self-efficacy in T1D conditions with family social support results in greater emotional wellbeing in children and adolescents with this disease. If there is strong family social support, the belief about disease control (self-efficacy) is positive and, therefore, the patient perceives greater emotional wellbeing, buffering the stress of living with the disease [44]. It appears sensible that fostering environments that instill confidence in individuals regarding their own disease would facilitate the adoption of healthy behaviors that prove beneficial in managing their condition [23]. In our study, the nuclear family emerges as a primary determinant of social support from friends or another significant individual, and serves as a reference point for young people with T1D. This aligns with the significance of our immediate social circle in achieving emotional balance, as it encompasses feelings of belonging, or of being included, supported, or guided [14], and also aligns with other studies that suggest that greater perceived social support from the family increases the subjective wellbeing of young people [45].

On the other hand, our structural equation modeling also reveals that positive expectations regarding health management may be contingent upon the emotional subjective wellbeing of children and adolescents with T1D. Moreover, these expectations serve as predictors of the level of self-efficacy in disease control, which in turn predicts the emotional subjective wellbeing of patients. The role of self-efficacy appears as a predictor of emotional subjective wellbeing, as has been previously suggested in studies involving healthy adolescents [26,28].

Psychoeducational interventions could be effective in improving the quality of life of children and adolescents with T1D [46,47]. Although self-efficacy-focused education seems to have a potential to enhance the quality of life for individuals with diabetes, further high-quality research is needed to substantiate these findings [48]. However, with our results, we must highlight the opportunities there could be for psychoeducational interventions to focus on enhancing self-efficacy in the improvement of emotional subjective wellbeing among this population. Indeed, in the context of endocrinology patients with diabetes, individuals who possess higher levels of self-efficacy in managing their condition are more likely to experience better overall wellbeing, and a higher quality of life [29,30]. To do so, it is recommended to incorporate self-care strategies aimed at improving self-regulation in adolescents with T1D [49]. Moreover, we also note the potential power of working with parents and emphasizing the importance of their support; for example, through empowerment in the management of their child’s illness [44].

### Limitations and Future Perspectives

Although the sample size aligns with similar published studies, and is representative of this specific clinical population, it would be valuable to increase the sample size further; for instance, through a multicenter investigation. Moreover, as we have continued with data collection while preparing this manuscript, the sample size has slightly increased. These additional data could provide greater consistency to our study in future works, if the results continue to align in the same direction. It also appears necessary to further evaluate the changes experienced by children and adolescents with T1D in perceived wellbeing and the self-regulatory variables that we have incorporated in this study; to assess the changes, it would be interesting both to be able to evaluate the stressful events they face, and the efficacy of psychoeducational interventions. Another limitation is the omission of biomedical variables. In future research, it is crucial that biomedical variables such as HbA1c levels or time in range be included, to truly assess the impact of psychosocial variables, particularly self-efficacy and emotional subjective wellbeing, in pediatric and young patients with T1D. Additionally, exploring psychosocial variables in parents could also contribute to a more comprehensive understanding of the current state of research in this field.

## 5. Conclusions

Our study provides an explanatory model on emotional subjective wellbeing in children and adolescents with T1D, with a longitudinal design, highlighting the pivotal role of family social support in their self-efficacy in managing the disease, and their emotional subjective wellbeing. These findings emphasize the significant implications of family social support on fostering emotional subjective wellbeing, and enhancing self-care capabilities. In addition, self-efficacy for managing T1D acts as a mediator between family social support and emotional subjective wellbeing. Future psychoeducational interventions aimed at emotional wellbeing in this context should explicitly address self-efficacy for disease management, and supportive environments for children and adolescents with T1D and their parents.

## Figures and Tables

**Table 1:** Demographic and clinical data of the total study sample, and differentiated by sex.

Demographic and Clinical Data	T1	T1_boys_	T1_girls_	T2	T2_boys_	T2_girls_
N (%)	151	88 (58.30)	62 (41.10)	97	59 (60.80)	38 (39.20)
Age (*Mean*, *SD*)	14.50 (2.67)	14.41 (2.55)	14.58 (2.83)	14.93 (2.56)	14.76 (2.26)	15.18 (2.98)
Age at diagnosis (*Mean*, *SD*)	8.65 (4.18)	9.00 (4.19)	8.21 (4.17)	8.82 (4.21)	9.03 (4.13)	8.50 (4.37)
Duration of illness (*Mean*, *SD*), years	5.90 (4.28)	5.55 (4.27)	6.29 (4.23)	6.95 (4.39)	6.44 (4.11)	7.74 (4.75)
Regimen						
-Basal-bolus injections (*n*, %)	137 (90.70)	81 (92.00)	55 (88.70)	88 (90.70)	55 (93.20)	33 (86.80)
-Insulin infusion pump (*n*, %)	14 (9.30)	7 (8.00)	7 (11.30)	9 (9.30)	4 (6.80)	5 (13.20)
Time in range (*Mean*, *SD*), %	55.68 (18.45)	55.77 (20.08)	55.37 (16.29)	55.73 (18.67)	55.43 (19.45)	56.23 (17.62)
HbA1c (*Mean*, *SD*)	7.41 (1.29)	7.34 (1.37)	7.51 (1.18)	7.68 (1.57)	7.79 (1.83)	7.49 (1.07)

Note. One patient identified as non-binary in T1. The time in range and HbA1c were only analyzed when the data were available in the medical report. For the time in range, *n* = 122 (57.38% boys) at T1 and *n* = 79 (62.03 boys) at T2. For HbA1c, *n* = 93 (61.59% boys) at T1 and *n* = 64 (60.94% boys) at T2. *SD*, standard deviation.

**Table 2:** Descriptive statistics and correlations (Spearman’s Rho) at T1 between emotional subjective wellbeing and all variables included in the study.

Variables	1	2	3.1	3.2	3.3	4.1	4.2	4.3	5
1. Age	1								
2. Self-efficacy DM Manag.	−0.04	1							
*Outcomes expectations DM*									
3.1. OEDM-P_Social	−0.21 **	0.33 ***	1						
3.2. OEDM-P_Energy	0.01	0.15	0.37 ***	1					
3.3. OEDM-P_Health	−0.10	0.24 **	0.38 ***	0.45 ***	1				
*Perceived social support*									
4.1. MSPSS_Friends	−0.16 *	0.52 ***	0.44 ***	0.32 ***	0.27 ***	1			
4.2. MSPSS_Family	0.02	0.28 ***	0.30 ***	0.06	0.20 *	0.32 ***	1		
4.3. MSPSS_Significant	0.06	0.30 ***	0.29 ***	0.28 ***	0.32 ***	0.57 ***	0.47 ***	1	
5. Emotional Subj.Wellbeing	−0.19 *	0.51 ***	0.32 ***	0.20 *	0.33 ***	0.55 ***	0.40 ***	0.47 ***	1
*M*	14.50	3.97	3.42	4.19	4.51	4.29	4.40	4.41	2.19
*SD*	2.66	0.68	1.06	1.00	0.56	0.81	0.75	0.69	1.41
*S*	−0.14	−0.66	−0.23	−1.17	−1.60	−1.22	−1.92	−1.86	−0.92
*K*	−1.16	−0.08	−0.92	0.52	3.30	0.67	4.56	4.15	0.48
*K-S*	0.13 ***	0.09 **	0.09 **	0.26 ***	0.21 ***	0.19 ***	0.21 ***	0.20 ***	0.14 ***

Note. Data of T1 (N = 151). * *p* < 0.05; ** *p* < 0.01; *** *p* < 0.001. SEDM-SF, Self-Efficacy for Diabetes Self-Management Short Form; OEDM-P, Positive Outcomes Expectations of Diabetes Self-Management; MSPSS, Multidimensional Scale of Perceived Social Support; ESW, emotional subjective wellbeing; *M*, mean; *SD*, standard deviation; *S*, skewness; *K*, kurtosis; *K-S*, Kolmogorov–Smirnov.

**Table 3:** Descriptive statistics and correlations (Spearman’s Rho) at T2 between emotional subjective wellbeing and all variables included in the study.

Variables	1	2	3.1	3.2	3.3	4.1	4.2	4.3	5
1. Age	1	−0.03	0.07	0.09	0.07	0.02	0.18	0.15	−0.23 *
2. Self-efficacy DM Manag.		1	0.34 ***	0.24 *	0.38 ***	0.58 ***	0.45 ***	0.41 ***	0.47 ***
*Outcomes expectations DM*									
3.1. OEDM-P_Social			1	0.59 ***	0.42 ***	0.37 ***	0.33 ***	0.29 **	0.23 *
3.2. OEDM-P_Energy				1	0.57 ***	0.40 ***	0.15	0.25 *	0.27 **
3.3. OEDM-P_Health					1	0.39 ***	0.32 ***	0.49 ***	0.44 ***
*Perceived social support*									
4.1. MSPSS_Friends						1	0.47 ***	0.67 ***	0.48 ***
4.2. MSPSS_Family							1	0.55 ***	0.45 ***
4.3. MSPSS_Significant								1	0.37 ***
5. Emotional Subj.Wellbeing									1
*M*	14.93	3.90	3.36	4.08	4.38	4.30	4.30	4.39	2.09
*SD*	2.56	0.73	1.13	1.12	0.79	0.83	0.78	0.73	1.51
*S*	−0.02	−0.54	−0.22	−1.30	−1.96	−1.63	−1.20	−1.46	−0.97
*K*	−1.21	−0.30	−0.93	0.96	4.59	2.60	1.29	2.25	0.90
*K-S*	0.13 ***	0.12 ***	0.10 *	0.21 ***	0.22 ***	0.20 ***	0.19 ***	0.20 ***	0.11 ***

Note. Data of T2 (N = 97). * *p* < 0.05; ** *p* < 0.01; *** *p* < 0.001. SEDM-SF, Self-Efficacy for Diabetes Self-Management Short Form; OEDM-P, Positive Outcomes Expectations of Diabetes Self-Management; MSPSS, Multidimensional Scale of Perceived Social Support; ESW, emotional subjective wellbeing; *M*, mean; *SD*, standard deviation; *S*, skewness; *K*, kurtosis; *K-S*, Kolmogorov–Smirnov.

## Data Availability

Not applicable.
